# Attack and defense networks in a student social system

**DOI:** 10.1371/journal.pone.0348814

**Published:** 2026-05-18

**Authors:** Alejandro Morales-Huitrón, Ana María Hernández-Hernández, Efrain Canto-Lugo, Rodrigo Huerta-Quintanilla

**Affiliations:** 1 Departamento de Investigación y Estudios Multidisciplinarios, Centro de Investigación y de Estudios Avanzados del Instituto Politécnico Nacional, Unidad Zacatenco, Av. IPN 2508, 07360, Ciudad de México, México; 2 Departamento de Física Aplicada, Centro de Investigación y de Estudios Avanzados del Instituto Politécnico Nacional, Unidad Mérida, Km 6 Carretera Antigua a Progreso, 97310, Cordemex, Mérida, Yucatán, México; University of Glasgow, UNITED KINGDOM OF GREAT BRITAIN AND NORTHERN IRELAND

## Abstract

Signed networks are a tool that researchers use to study the relationships between individuals in a complex system. Many studies focus on negative relationships and how these shape the structure of complex networks. Negative hubs, or nodes with the most negative connections, are of particular interest to researchers, as they help us understand social phenomena such as bullying, cyberbullying, and mental health. In this work, we study directed signed networks that represent positive (friendship) and negative (enmity) relationships between students of different academic levels. We propose a systematic methodology to obtain and analyze the networks of nine schools (∼4,300 students) in Yucatán, México. We introduce attack and defense subnetworks constructed from nodes with high out-negative and in-negative degree, respectively, and examine whether these subnetworks exhibit similar structural properties. Using the Leiden algorithm, we detected directed signed communities and compared the community structure of the attack and defense networks. We also calculated social balance theory measures and discussed the outcomes we obtained. We identify a range within which the definition of attack and defense networks is structurally meaningful. We have observed that the structural properties of attack and defense networks are similar and that they are not mutually exclusive, with an average of 60% common nodes, indicating a strong overlap between the two networks. Also, we have evaluated the attack and defense networks in relation to the gender of students, and found that female networks were generally denser across schools. Finally, we discuss the conceptual relationship between these networks and bullying-related roles.

## Introduction

Network theory helps us study the relationships (links/ties) between different elements (nodes) inside a system [1]. When the system under study is merged in a social context, these are referred to as social networks [2].

Traditionally, most studies have focused on simple networks, that is, networks consisting only of undirected positive links [3]. More recently, researchers have begun to consider signed networks [4–6] and directed signed networks [7]. Directed signed networks extend simple networks by allowing each link to carry both a direction and a sign (positive or negative) [8]. In this work, we analyze directed signed networks in school environments, where positive and negative links indicate whether students have friendship or enmity relationships, respectively.

Communities are an important concept in network theory and are defined as a group of nodes that are densely connected to each other but sparsely connected to other nodes in the network [9]. In a school network, it is expected that nodes within the same community share hobbies, habits, or similar ideas, while nodes from different communities may have fewer affinities or even opposing views [10]. Positive (friendship) links tend to reinforce cohesion within communities, whereas negative (enmity) links often discourage such cohesion and may form across community boundaries [11]. The process of community detection in networks is itself a subject of study in the field of complex networks. There are several algorithms with different success rates for detecting communities. Many of these algorithms aim to maximize an objective function called modularity [12]. These algorithms use links as attractive forces to build communities [13]. When dealing with signed networks, these algorithms are generalized to incorporate negative links as repulsive forces [13]. In the case of directed signed networks, this generalization also takes into account the direction of the links [14]. On the other hand, social balance is a network-level property that arises from signed networks and is used to assess the stability or tension within them, based on positive and negative relationships among triads of nodes [15,16].

Recently, many studies have analyzed the importance of negative links in social networks and how they determine the topology and other properties of networks [13,17–19]. In accordance with this research line, in this study we investigate the directed signed networks of face-to-face relationships among students from nine schools at different academic levels, and we focus on nodes with the highest negative connectivity (negative hubs). These nodes have been shown to play an important role in the organization of signed networks [6].

We define the attack and defense networks as subnetworks constructed from out-negative hubs and in-negative hubs respectively, and we define a range (*p*) where their definitions are structurally meaningful. Because negative ties in directed networks are asymmetric, distinguishing out-negative hubs from in-negative hubs separates nodes that primarily emit negative relationships from those that primarily receive them. This subnetwork-based approach provides a meso-level perspective that isolates the core of antagonistic relations while preserving both positive and negative ties among selected nodes, enabling a more interpretable assessment of density, social balance, and signed community structure. To the best of our knowledge, this type of subnetwork construction has not been previously explored in the context of directed signed networks. This represents a novel methodological contribution that enables the analysis of mesoscale structures centered on antagonistic interactions.

In this study, rather than comparing attack or defense subnetworks to the full network, our goal is to evaluate whether the subnetworks themselves share similar topological properties. By comparing attack and defense subnetworks across multiple schools and node-selection thresholds, we assess whether antagonistic senders and receivers form structurally similar mesoscale patterns. Specifically, our research question is: Do attack and defense subnetworks exhibit similar structural, community, and balance properties across school networks?

This study is primarily descriptive, with a methodological component, as it introduces a systematic approach to construct and analyze subnetworks obtained from the most negative hubs. In the following section (Materials and Methods), we describe the data collection process carried out in the schools studied and define the concepts of attack and defense networks. We also expand on the descriptions of modularity and social balance, including their mathematical formulations. These two measures will help support some conclusions drawn from this study.

## Materials and methods

### Data collection

The dataset used in this study was formally published in a peer-reviewed journal and is available in the supporting information section of the original article [19]. While the dataset was previously published, the analyses, research question, methodology, and conclusions presented here are entirely new and therefore do not constitute dual publication. We accessed the data for research purposes on March 5, 2024. Originally the dataset was collected through the application of electronic surveys administered during school hours under teacher supervision. During data collection, we had temporary access to information that could identify individual participants. The process was personally supervised by our research group after providing a brief explanation of the study objectives. All data were handled confidentially and analyzed in anonymized form. Details regarding ethical approval are provided in the study where the dataset was originally published.

The questionnaire (survey) consisted of two parts: the first, with six questions corresponds to general student data, and the second part with seven questions assess data on friendship, enmity, and kinship relationships among students at the same school. Participation was voluntary, and students could freely nominate any of their schoolmates for each relational question (friendship, enmity, and kinship) without restrictions on the number of nominations. To avoid conflict or misunderstanding, the term ‘enmity’ was replaced by ‘bad relationships’ in the questionnaire. The response rate across the nine schools ranged between 95% and 97% of the enrolled students. Only gender (personal data, question 3) and friendship/enmity nominations (social data, questions 3 and 4) were used in the present study. The questionnaire is attached in support information (S1 File). The dataset obtained from these surveys has also been used in research [20]. Table 1 shows the characteristics of the nine schools relevant to the study.

**Table 1:** Characteristics of the schools that participated in this study.

School	Degree	Age Range	Students	Males	Females
esSC	Elementary	6y to 12y	108	56	52
esRRC	Elementary	6y to 12y	226	108	118
esIZ	Elementary	6y to 12y	419	200	219
ssRDC	Secondary	12y to 15y	613	302	311
ssTN2	Secondary	12y to 15y	457	226	231
ssJLBG	Secondary	12y to 15y	270	132	138
hsCCP	High	15y to 18y	1497	743	754
hsHUN	High	15y to 18y	74	37	37
usTRS	University	18y to 22y	664	360	304

In México, secondary school and high school students are taught in different schools.

Using the information obtained from the surveys, the nine corresponding adjacency matrices (*A*) were constructed. These matrices are signed and non-symmetric, and they represent the nine directed signed networks (hereafter referred to as original networks). In these matrices, *A*[*i*,*j*] = 1 indicates a directed friendship from node *i* to node *j*, *A*[*i*, *j*] = −1 indicates a directed enmity from node *i* to node *j*, and *A*[*i*,*j*] = 0 indicates no relationship between the nodes. Adjacency matrices are included in S1 Dataset. In addition, S2 Dataset contains an anonymized node-level table including node identifiers, gender, and classroom membership for all schools analyzed.

### Attack and defense networks

On undirected signed networks, many studies are focused on analyzing negative subnetworks, often referred to as dislike networks. [8,21]. The most negatively connected nodes, known as negative hubs, are particularly significant. Understanding their properties, roles, and relationships with one another is of great interest [22].

In this study, we analyze directed signed networks and define attack and defense networks as follows. Attack networks are the subnetworks formed by the most negatively connected nodes, considering only their outgoing links (out‑negative hubs). Likewise, defense networks are the subnetworks formed by the most negatively connected nodes, but considering only their incoming links (in‑negative hubs). The number of nodes that constitute the attack and defense networks corresponds to a given percentage (*p*) of the nodes in the original directed signed network. The construction of the subnets is now described step by step.

1. Directed signed degrees. For each original network, we compute the out- and in-negative degrees of every node: $k_{out}^{-}(i)=\sum_{j}1[A_{ij}=-1]$ and $k_{in}^{-}(i)=\sum_{j}1[A_{ji}=-1]$.
2. Ranking and target size. For a target percentage p∈{20%,25%,30%,35%,40%}, we set the subnetwork size $n_{p}=\lfloor p*N\rfloor$.
3. Attack network (out-negative hubs). We sort nodes by $k_{out}^{-}$ (descending). If multiple nodes have the same $k_{out}^{-}$ at cut-point (percentage *p*), we randomly sample without replacement from the tied set to reach *n*_*p*_. We then induce the directed signed subgraph on the selected nodes, retaining both positive and negative links among them.
4. Defense network (in-negative hubs). Identical procedure but sorting by $k_{in}^{-}$.
5. Stochastic replication. Because ties occur frequently, each (school, *p*) condition is replicated 500 times.
6. Aggregation. For each metric (e.g., $\langle k\rangle^{+}$, $\langle k\rangle^{-}$, *Q*^*s*^, number of communities, social balance), we report the mean across the 500 replicas.

The average degree ⟨k⟩ of a directed signed network represents the mean number of links per node and can be computed separately for positive and negative relationships as $\langle k\rangle^{+}=m^{+}/N$ and $\langle k\rangle^{-}=m^{-}/N$, where *m*^+^ and *m*^−^ denote the total numbers of positive and negative directed links, respectively, and *N* is the number of nodes in the network. These values quantify, on average, how many friendship (positive) or enmity (negative) relationships each student reports within the network.

**Fig 1:**
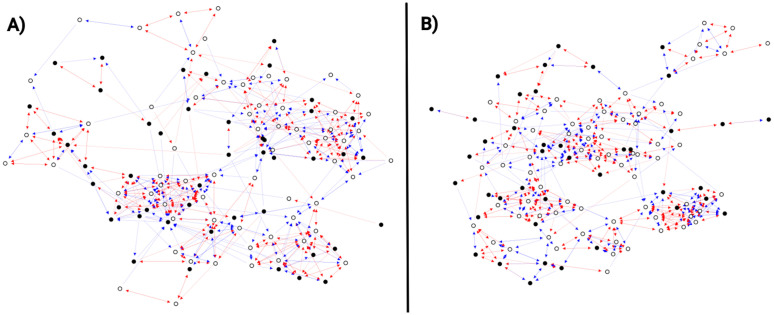
Attack and defense networks at *p =* 25% of school ssTN2. **(A)** Attack network with 113 nodes (65 females and 48 males) and 568 links (240 positive and 328 negative). **(B)** Defense network with 111 nodes (68 females and 43 males) and 572 links (242 positive and 330 negative). The color of the nodes represents the gender of the students: males (black) and females (white). The color of the links indicates the type of relationship between the nodes: friendship (blue) and enmity (red).

Fig 1 shows an attack network and a defense network from school ssTN2 (457 students). Both networks are constructed using *p* = 25% of the nodes from the ssTN2 network. In Fig 1A, the attack network is shown with 113 nodes and 568 links. This network is formed by the relationships of the out-negative hubs, i.e., the nodes with the highest $k_{out}^{-}$ values in the ssTN2 network. Both positive (blue links) and negative (red links) relationships between the nodes are displayed. The average degrees are $\langle k\rangle^{+}=2.12$ and $\langle k\rangle^{-}=2.90$. Fig 1B shows the defense network with 111 nodes and 572 links. This network includes the nodes with the highest $k_{in}^{-}$ values (in-negative hubs) and their positive and negative relationships. The average degrees are $\langle k\rangle^{+}=2.18$ and $\langle k\rangle^{-}=2.97$. In both cases, the average degree values indicate that there are more negative than positive relationships in the networks. The figure also shows that the nodes in the attack and defense networks do not necessarily retain their negative hub status (an attribute they have in the original network); there are nodes with few negative links and even some with only positive links. Finally, this example shows a slight difference in the number of nodes between the two networks, which results from the randomness introduced during their construction.

We focus on p∈[20%,40%] based on three empirical criteria observed consistently across schools and attack and defense subnetworks:

- Structural equilibrium. For p∈[20%,40%], attack and defense subnetworks maintain a structural equilibrium between external and internal connectivity.
- Statistical stability. For *p* < 20%, subnetworks become too small for stable estimation of degrees, modularity, and transitive triads. At *p* = 20%, negative links dominate and sufficiently stable subnetworks are formed (connected and dense), which preserves the interpretability of negative-hub centered structures.
- Conceptual specificity. For *p* > 40%, positive links increasingly dominate and many included nodes no longer qualify as negative hubs, diluting the construct the subnetworks are meant to capture.

The concept of structural equilibrium was first introduced by Krackhardt and Stern [23] and refers to the relationship between external and internal connectivity within a subnetwork embedded in a larger network. A structurally balanced subnetwork is neither isolated from the global system nor completely absorbed by it. Instead, it maintains sufficient internal ties to sustain a distinct structural identity, while also preserving enough external connections to remain functionally integrated into the overall network. The classical formulation is given by E|I=(E−I)/(E+I), where *E* denotes the number of external links and *I* the number of internal links within the subnetwork. This measure ranges from [−1, 1], where values close to −1 indicate a relatively isolated subnetwork, and values close to 1 indicate subnetworks that are overly integrated into the global system. A normalized version of this measure is given by φ=E/(E+I), which ranges from [0, 1], with intermediate values indicating structural equilibrium. Because our subnetworks are constructed based on negative links, we focus on assessing structural equilibrium with respect to these ties. For the attack networks, we use Eq. 1:


$$\begin{array}{c} \varphi_{out}^{-}=\frac{E_{out}^{-}}{E_{out}^{-}+I_{out}^{-}} \end{array}$$ (1)

where $E_{out}^{-}$ denotes the external out-negative links and $I_{out}^{-}$ denotes the internal out-negative links within the subnetwork. The formula for defense networks ($\varphi_{in}^{-}$) is defined analogously. Following the notion of structural equilibrium proposed by Moody and White [24], meaningful subnetworks are expected to exhibit a balance between internal cohesion and external connectivity. Empirically, this balance is often observed within intermediate ranges of external-to-total connectivity, commonly between 0.3 and 0.7, where subnetworks remain internally coherent while still embedded in the broader network structure [25, 26]. We computed the values of $\varphi_{out}^{-}$ and $\varphi_{in}^{-}$ (see Fig A in S1 Fig) and found that, within the range p∈[20%,40%] both attack and defense networks satisfy the criterion of structural equilibrium.

**Fig 2:**
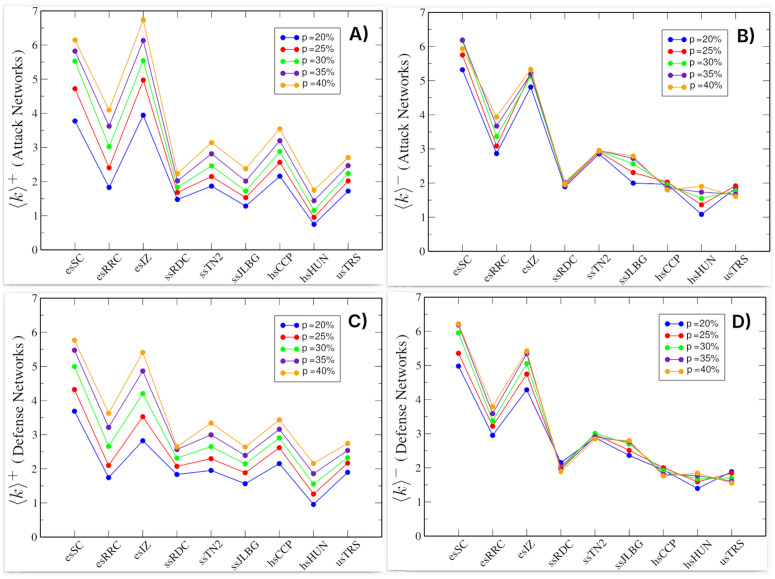
Average degree of the attack and defense networks in the nine schools studied. **(A)** and **(B)** Attack networks: average degree for positive and negative links, respectively. **(C)** and **(D)** Defense networks: average degree for positive and negative links, respectively. Color scale indicates percentage of nodes (*p*) included from the original network. Each value was obtained after 500 simulations.

We also empirically verified the criteria for statistical stability and conceptual specificity by inspecting the average degree values ($\langle k\rangle^{+}$ vs $\langle k\rangle^{-}$) trajectories across *p*. Fig 2 shows the average degree values for the attack networks (top) and defense networks (bottom). On the left are the values of $\langle k\rangle^{+}$ and on the right $\langle k\rangle^{-}$, representing the average number of positive and negative links, respectively. In both attack and defense networks, we observe that at *p* = 20%, there are more negative links than positive ones. As the percentage increases, the difference in favor of negative links decreases. At *p* = 40%, the number of positive and negative links becomes approximately equal; in some cases, the networks even have more positive links. We also analyzed the values above 40% and observed that the number of positive links increased considerably. This is because beyond *p* = 40%, the number of negative links remains nearly constant, while the number of positive links continues to increase. This behavior is also evident in Fig 2. Furthermore, we observed that for values above 40%, nodes would be included in attack and defense networks that can no longer be considered negative hubs, due to the low number of negative links they have.

Finally, we adopt the terms attack and defense networks because, to the best of our knowledge, there is no established terminology in complex network theory for the type of subnetworks defined in this subsection.

### Leiden algorithm

In the literature, a community is defined as a group of nodes that are densely connected to each other and sparsely connected to the rest of the network [9]. Community detection is a very important area in the study of social networks [27,28]. This process is carried out using computational algorithms. A method that is widely used is the Louvain algorithm [29,30], which creates a partition of the network into communities. The number and size of the communities are not predetermined; instead, they are dictated by the structure of the network itself. The goal of the algorithm is to produce an optimal community partition. To achieve this, it uses an objective function called modularity (*Q*^+^), which compares the fraction of positive links within communities to that expected in a topologically similar random network.

Community detection in signed networks is a current topic of interest in the field of complex networks [14,31–33], where algorithms aim to create groups of nodes with positive connections within the same community and negative connections between different communities. The Louvain algorithm has been extended to compute modularity in signed networks. For this purpose, two new concepts are introduced: negative modularity (*Q*^−^), which is defined analogously to positive modularity; and signed modularity (*Q*^*s*^), which is defined as a linear combination of *Q*^+^ and *Q*^−^. In other words, *Q*^*s*^ represents the trade-off between positive links contributing to community formation and negative links working against it [19].

The Leiden algorithm is an improvement over the Louvain algorithm, offering better-connected communities and greater computational efficiency. There exists an implementation of this method for detecting communities in directed signed networks, which is the version used in this work. A detailed description of the Leiden method can be found in [34]. This method also optimizes the positive modularity function (*Q*^+^), but takes into account the direction of the links, as described in Eq. 2.


$$\begin{array}{c} Q^{+}=\frac{1}{m^{+}}\sum_{c}\left(m_{c}^{+}-\frac{k_{in,c}^{+}\cdot k_{out,c}^{+}}{m^{+}}\right) \end{array}$$ (2)

where *m*^+^ represents the number of directed positive links in the network. Here, *C* denotes the set of communities detected by the algorithm, and the summation runs over each community c∈C. $m_{c}^{+}$ refers to the directed positive links in community *c*, and the parameters $k_{in,c}^{+}$ and $k_{out,c}^{+}$ denote the sums of positive in-degree and out-degrees over nodes in community *c*, respectively. The function *Q*^−^ is defined analogously, but for directed negative links. Thus, the signed modularity function (*Q*^*s*^) is defined as shown in Eq. 3.


$$\begin{array}{c} Q^{s}=\frac{m^{+}}{m^{+}+m^{-}}Q^{+}-\frac{m^{-}}{m^{+}+m^{-}}Q^{-} \end{array}$$ (3)

Networks with a high *Q*^*s*^ value will have many directed positive links within communities and few directed positive links between them. In addition, they will have few directed negative links within communities and many directed negative links between them. Note that in the absence of directed negative links, $Q^{s}=Q^{+}$. A detailed formulation of *Q*^*s*^ and *Q*^+^ can be found in [35].

#### Communities computation (directed signed).

In this work, we used the function find_partition_multiplex (Leidenalg version 0.10.2), which allows detecting communities in graphs that contain multiple layers sharing the same set of nodes. In our particular case, the function returns an optimal partition of directed signed communities and receives four parameters:

- [g_pos, g_neg]. The attack and defense networks can be represented as two layers with exactly the same set of nodes, where g_pos is the layer containing only directed positive links and g_neg the layer containing only directed negative links.
- leidenalg.ModularityVertexPartition. Implements the Leiden algorithm for community detection in networks. This specific class represents a partition of a graph’s nodes into communities, optimized according to the modularity quality function.
- layer_weights=[1, −1]. This parameter specifies that the network is signed, with the values corresponding to the weights of the links in g_pos and g_neg, respectively.
- max_comm_size = 0. This parameter indicates that there is no restriction on the size of the detected communities.

The remaining parameters were set to their default values (technical details can be found in the official documentation [36]). Once the community partition was obtained, we calculated the values of *Q*^+^, *Q*^−^, and *Q*^*s*^ using the formulas described above. We report, for each (school, *p*) condition, the mean *Q*^*s*^ and mean number of communities across the 500 replicas.

### Social balance in directed signed networks

Social balance theory is an important topic in the field of signed networks. It considers the possible ways in which the links of a triad of nodes can be signed. This theory postulates that socially balanced triads should be more frequent in networks. A detailed description of this theory can be found in [37].

In the case of directed signed networks, when calculating social balance, the direction of the links must be taken into account. As a result, there are different types of node triads, which have been classified using a specific nomenclature. This theory also defines the concepts of transitive semi-cycles, balanced transitive triads, and unbalanced transitive triads. The details of these definitions and the corresponding nomenclature can be found in [7].

#### 2.4.1. Social balance computation (directed signed).

Below we describe the steps to calculate the social balance in attack and defense networks. Here, social balance is assessed in terms of transitive balance triads, following standard definitions for directed signed networks.

1. Triads analyzed. We restrict to transitive triads with nomenclature 030*T*, 120*D*, 120*U*, and 300 (the types with the largest number of transitive semi-cycles), see Fig B in S1 Fig.
2. Per-simulation pipeline. Using networkx 2.8.8 library [38], we (i) census all directed triads, (ii) identify transitive triads of the above types, (iii) decompose each into its transitive semi-cycles, and (iv) label each semi-cycle as balanced if the sign product is positive. A triad is balanced if all its transitive semi-cycles are balanced.
3. Social balance index. SB = ${△}_{t}^{b}/({△}_{t}^{b}+{△}_{t}^{ub})$, where ${△}_{t}^{b}$ and ${△}_{t}^{ub}$ are the counts of balanced and unbalanced transitive triads, respectively.
4. Aggregation and reporting. For each (school, *p*), we compute SB in each of the 500 replicas and report the mean SB.

## Results

### Properties of the directed signed networks

We begin by characterizing the distribution of positive and negative relationships across schools at different academic levels. Table 2 shows the properties of the nine directed signed networks (original networks) analyzed in this study. The information provided includes the number of nodes and the corresponding directed positive (*m*^+^) and negative (*m*^−^) links for each school. $\langle k\rangle^{+}$ and $\langle k\rangle^{-}$ represent the average degrees of friendship and enmity, respectively. In addition, the *m*^−^ column shows the percentage of negative links in each school.

**Table 2:** Positive and negative links in the directed signed networks.

Network	Nodes	*m* ^+^	*m*^−^ (%)	$\langle k\rangle^{+}$	$\langle k\rangle^{-}$
esSC	108	1220	607 (33.2%)	11.3	5.6
esRRC	226	2397	1125 (31.9%)	10.6	5.0
esIZ	419	5377	2363 (30.5%)	12.8	5.6
ssRDC	613	2994	983 (24.7%)	4.9	1.6
ssTN2	457	2840	948 (25.0%)	6.2	2.1
ssJLBG	270	1523	588 (27.8%)	5.6	2.2
hsCCP	1497	11618	1650 (12.4%)	7.8	1.1
hsHUN	74	367	122 (24.9%)	5.0	1.6
usTRS	664	3582	694 (16.2%)	5.4	1.0

Columns *m*^+^ and *m*^−^ represents positive and negative directed links number respectively. $\langle k\rangle^{+}$ and $\langle k\rangle^{-}$ are the friendship and enmity directed links per node.

Table 2 also shows that in the three elementary schools (esSC, esRRC, esIZ), the proportion of negative links is in the range of (30% − 34%). In the three secondary schools (ssRDC, ssTN2, ssJLBG), this proportion is in the range of (24% − 28%). For the high schools and university, there is a lower proportion of negative relationships, except for hsHUN, which might be due to the small number of students in that school. Although hsHUN is a network with few nodes, we decided to include it in this study to determine whether it behaves similarly to the other networks or displays atypical properties.

Although elementary schools have a lower proportion of positive links, their $\langle k\rangle^{+}$ values are higher than those of the other schools; the same occurs with the $\langle k\rangle^{-}$ values. This indicates that the elementary school networks are denser than the others. Students aged 6–12 report more friendships but also more enmities at school. For students 12 and older, the number of friendships and enmities with other students decreases considerably, and this phenomenon occurs regardless of the size of the system. It is also interesting to note that the ssRDC and hsHUN networks, two schools with very different characteristics (see Table 1), have very similar properties.

### The i/o-negative hubs

In this study, we observed that a considerable proportion of students belong to both attack and defense networks, indicating a substantial overlap between in-negative hubs and out-negative hubs. We refer to these nodes as i/o-negative hubs.

**Fig 3:**
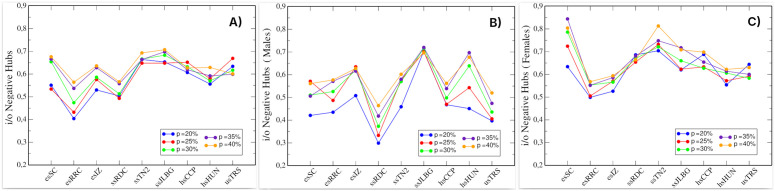
Proportion of nodes belonging simultaneously to both attack and defense networks. **(A)** Proportion of i/o-negative hubs. **(B)** Proportion of i/o-negative hubs (male networks). **(C)** Proportion of i/o-negative hubs (female networks). Color scale indicates percentage of nodes (*p*) included from the original network. Each value was obtained after 500 simulations.

Fig 3 displays the proportion of i/o-negative hubs for each (school, *p*) condition. This proportion was calculated as the ratio between the number of nodes that appear simultaneously in both the attack and defense networks and the total number of unique nodes in these two subnetworks. This measure represents the extent to which out-negative and in-negative hubs overlap. As shown in Fig 3A, when attack and defense networks are formed using *p* = 20% of the nodes from the original networks (blue line), at least half of the nodes in nearly all schools are i/o-negative hubs. As the size of the attack and defense networks increases, the proportion of i/o-negative hubs also increases, reaching approximately 0.7. In other words, the orange line indicates that when the attack and defense networks include *p* = 40% of the original nodes, around 70% of the students belong to both networks simultaneously.

To examine gender-specific patterns among i/o-negative hubs, we replicated our analysis for attack and defense networks segregated by gender, selecting only male or female nodes from the original networks. Fig 3B presents results for male-only networks, where attack networks consist of male out-negative hubs and defense networks of male in-negative hubs. Similarly, Fig 3C presents the corresponding female-only networks, constructed from female out-negative and in-negative hubs, respectively.

It can be observed in Fig 3 that in all nine schools the female population shows higher proportions of i/o-negative hubs. This occurs even in schools where the male and female populations are statistically similar, as well as in schools with a majority male population. (see Table 1). Furthermore, averaging the respective graphs from Fig 3B and 3C yields values that closely match those of Fig 3A. This indicates that, although the male and female networks were constructed independently, the trends in their graphs complement each other.

### Properties of attack and defense networks

Using Fig 2, we previously described the methodology used to determine the percentage range p∈[20%,40%] employed to construct the attack and defense networks. In this section, we analyze this figure in more detail. It shows the average degree values for each school. As mentioned previously, Fig 2A and Fig 2B correspond to the attack networks, while Fig 2C and 2D correspond to the defense networks.

The first noticeable aspect is the similarity between attack and defense networks, with panels A and C showing comparable $\langle k\rangle^{+}$ values, and panels B and D showing comparable $\langle k\rangle^{-}$ values. This similarity is partly expected due to the overlap of i/o-negative hubs. However, the persistence of similar $\langle k\rangle^{+}$ and $\langle k\rangle^{-}$ values despite the presence of non-overlapping nodes indicates that this resemblance cannot be attributed solely to shared nodes, but reflects broader structural similarities between the two subnetworks. This is more evident in the case of negative links, since the networks are constructed based on them. This suggests that the i/o-negative hubs and their relationships contribute structurally to the organization of attack and defense networks, as their presence is associated with the persistence of similar degree patterns across subnetworks. As shown in Fig 2, elementary school attack and defense networks exhibit a higher average negative degree compared to other schools, consistent with the pattern observed in the original networks (see Table 2).

### Gender-based analysis of attack and defense networks

The gender-based comparisons are presented descriptively, highlighting structural patterns in the networks. We observed that the attack and defense networks include a slightly higher proportion of females than males (see Fig C and Fig D in S1 Fig). To describe how this pattern appears in network structure, we computed the average degrees of gender-based networks, following the same methodology used for Fig 3B and 3C. Here we present descriptive results for negative relationships, while graphs of positive relationships are included in Fig E in S1 Fig.

**Fig 4:**
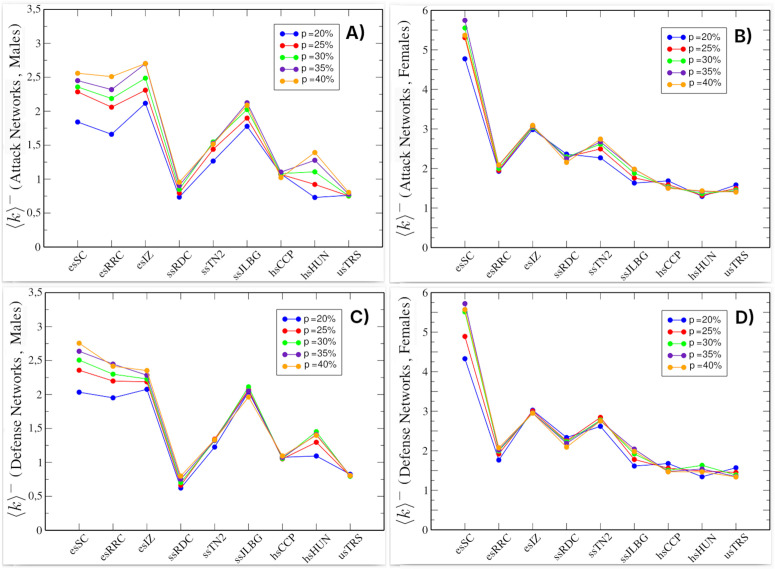
Average negative degree in attack and defense networks by gender. **(A)** Male attack networks (male out-negative hubs). **(B)** Female attack networks (female out-negative hubs). **(C)** Male defense networks (male in-negative hubs). **(D)** Female defense networks (female in-negative hubs). Color scale indicates percentage of nodes (*p*) included from the original network. Each value was obtained after 500 simulations.

Fig 4 shows negative relationships in the attack networks (top) and defense networks (bottom), with the female networks exhibiting higher average degrees, indicating that they are denser networks. Across all schools, the $\langle k\rangle^{-}$ values reach a consistent upper limit (it stabilizes), particularly in the female networks, where differences by percentage (*p*) are minimal. It is also notable that in the female networks (Fig 4B and 4D), the schools esSC, esIZ, and ssTN2 show the highest values, which could indicate the existence of conflicts between them. Fig 4 maintains the similarity pattern between $\langle k\rangle^{-}$ values in the attack and defense networks, more prominently in the case of females. This suggests that i/o-negative hubs and their relationships continue to contribute structurally to the organization of attack and defense networks, even when they are separated by gender.

### Analysis of attack and defense networks by relationship type

**Fig 5:**
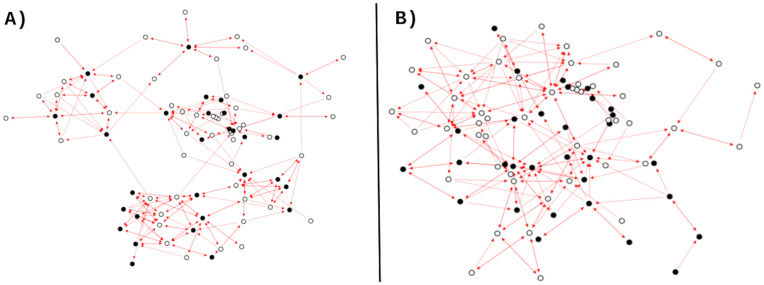
Negative link networks by relationship type. **(A)** Attack network of school esIZ at *p* = 20%. This network contains 81 nodes, of which 31 males and 50 females. Only negative relationships connecting cross-gender nodes are shown. **(B)** Defense network of school esIZ at *p* = 20%. This network contains 82 nodes, of which 31 males and 51 females. Only negative relationships connecting same-gender nodes are shown. The color of the nodes indicates the gender: males (black) and females (white). Note: The network in panel B has two disconnected components, as there are no links between male and female nodes.

We are now analyzing the attack and defense networks in terms of relationship type. We are interested in examining two distinct types of connections: those linking same-gender nodes and those linking cross-gender nodes. Fig 5 illustrates the negative connections between two types of nodes (males and females). In Fig 5A, we observe the negative relationships between cross-gender nodes in the attack network of school esIZ, constructed at *p* = 20%. Fig 5B shifts the focus to the defense network of the same school at *p* = 20%, highlighting the negative relationships between same-gender nodes. Positive links between the nodes of these same networks are included in Fig F in S1 Fig.

**Fig 6:**
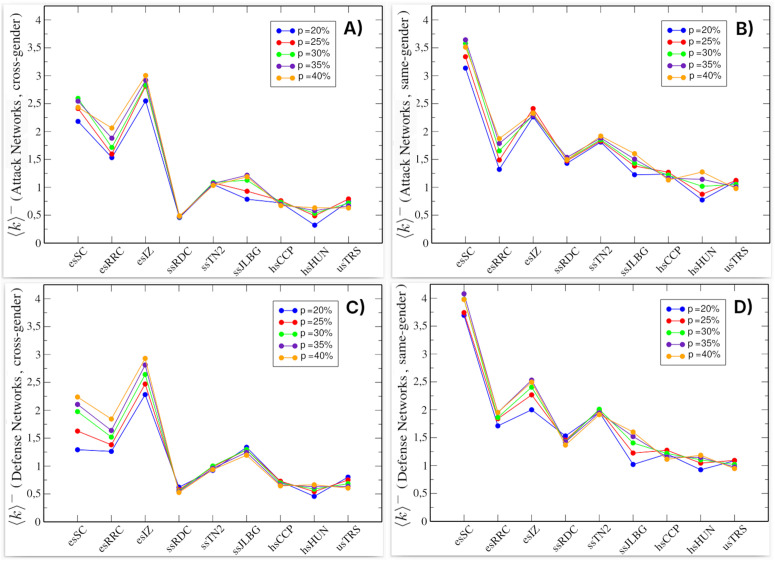
Average degree of negative links in attack and defense networks by relationship type. **(A)** Attack networks with relationships between cross-gender students. **(B)** Attack networks with relationships between same-gender students. **(C)** Defense networks with relationships between cross-gender students. **(D)** Defense networks with relationships between same-gender students. Color scale indicates percentage of nodes (*p*) included from the original network. Each value was obtained after 500 simulations.

As mentioned above, attack and defense networks generally include more females than males, and we are interested in analyzing how average degrees behave when relationships are separated by type, as illustrated in Fig 5. The average degrees of negative relationships are shown in Fig 6 (the results for positive relationships are included in Fig G in S1 Fig). Fig 6A and 6B (top) show the results for attack networks, while Fig 6C and 6D (bottom) show the results for defense networks. The graphs displaying cross-gender relationships between students (Fig 6A and 6C) clearly show that the three elementary schools (esSC, esRRC, and esIZ) have higher values, indicating that students report a greater number of negative relationships with cross-gender peers. In other words, there is some antagonism between boys and girls. For the rest of the schools, this pattern disappears, implying that enmity between males and females decreases.

In the case of same-gender relationships (Fig 6B and 6D), the values of $\langle k\rangle^{-}$ are higher compared to respective cross-gender relationships. Fig 6A and 6C, indicating greater enmity among same-gender students. A gradual decrease is also observed as school grade increases. As in previous graphs, all four panels show that the values of $\langle k\rangle^{-}$ stabilize, and in some schools these values are very close regardless of the percentage of nodes involved. Here again, the similarity in $\langle k\rangle^{-}$ values between attack and defense networks is evident. This suggests that the i/o-negative hubs and their relationships remain structurally important even when gender-specific relationships are analyzed separately.

### Social balance in attack and defense networks

So far, we have analyzed the positive and negative links independently, observing the behavior of the values $\langle k\rangle^{+}$ and $\langle k\rangle^{-}$ in the attack and defense networks. However, signed networks have properties and metrics that depend on both positive and negative links simultaneously. One of the most relevant properties in the field of signed networks is social balance, and in this section we examine how social balance behaves in the attack and defense networks. For this process, we restrict to transitive triads of types 030*T*, 120*D*, 120*U*, and 300, and assess whether they are balanced or unbalanced. To calculate the balance, we use the procedure described in the subsection ’Social balance computation’.

**Fig 7:**
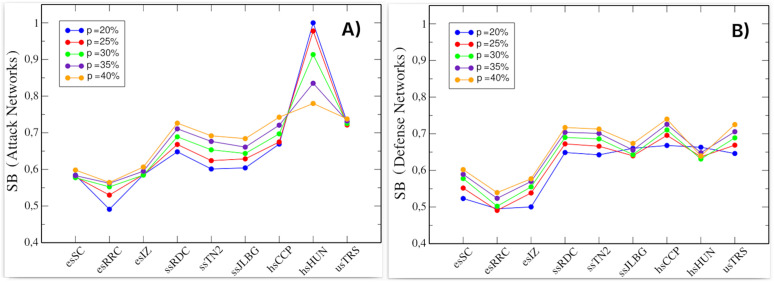
Social balance (SB) in attack and defense networks. **(A)** Social balance in the attack networks. **(B)** Social balance in the defense networks. Color scale indicates percentage of nodes (*p*) included from the original network. Each value was obtained after 500 simulations.

Fig 7 shows the behavior of social balance in the networks. In Fig 7A, we analyze the attack networks, revealing a consistent increase in balance values as the proportion of nodes increases (observed in all cases except for school hsHUN). This trend suggests a steady progression, though usTRS exhibits similar values across different *p* values. Our calculations also indicate that the number of transitive triads (particularly balanced ones) increases as network size grows. In contrast, the school hsHUN displays a different pattern: its social balance at *p* = 20% is higher than at *p* = 40%. This behavior arises because the original hsHUN network contains few nodes, resulting in an exceptionally low number of transitive triads, both balanced and unbalanced. Consequently, hsHUN displays atypical social balance dynamics compared to the other schools.

In general, as the networks grow, the number of transitive triads, both balanced and unbalanced, increases at almost the same rate, with the proportion of balanced transitive triads slightly higher. For defense networks (Fig 7B), we observe a similar behavior: social balance values increase as the number of nodes in the networks increases, with a difference of approximately 0.1 between the networks at *p* = 20% and those at *p* = 40%. In this case, the school hsHUN does not exhibit such an evident atypical behavior. In contrast, all the defense networks in this graph show a homogeneous pattern. Notably, while the social balance metric incorporates both positive and negative links in its computation, we observe consistent patterns between attack and defense network values, except for hsHUN school.

### Modularity in attack and defense networks

Modularity is another crucial concept, especially for signed networks. The optimal division of networks into communities and the calculation of signed modularity (*Q*^*s*^) are of significant interest. Like social balance, signed modularity is influenced by both positive and negative links. In this section, we examine how attack and defense networks respond to the Leiden algorithm for community detection in directed signed networks.

**Fig 8:**
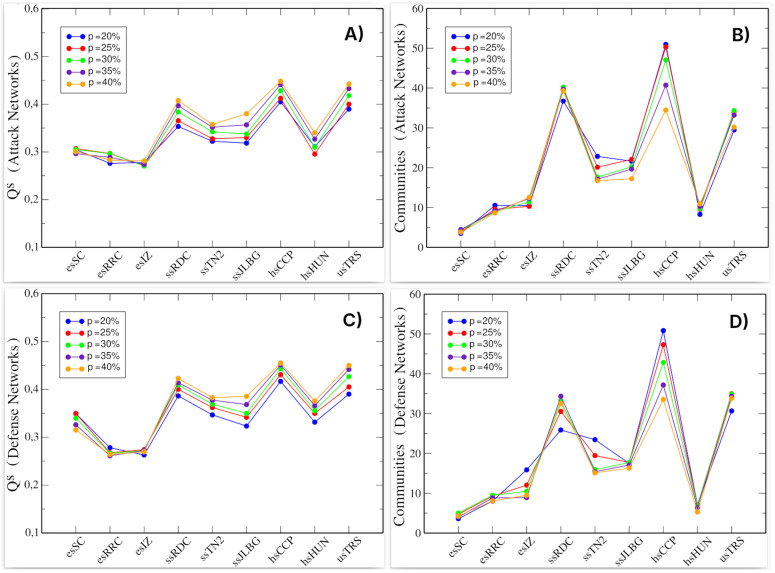
Signed modularity (*Q*^*s*^) and number of communities in attack and defense networks. **(A)** *Q*^*s*^ in the attack networks. **(B)** Number of communities in the attack networks. **(C)** *Q*^*s*^ in the defense networks. **(D)** Number of communities in the defense networks. Color scale indicates percentage of nodes (*p*) included from the original network. Each value was obtained after 500 simulations.

In Fig 8A and 8C, the *Q*^*s*^ values across all school networks are shown. The results reveal a general trend of increasing modularity with network size. However, elementary schools (esSC, esRRC, and esIZ) exhibit relatively stable modularity values, showing minimal variation across different network scales. This stability likely stems from their denser mixture of positive and negative links, which maintains community robustness even at the minimal network size (*p* = 20%). Rather than fragmenting, these networks maintain comparable community structure as they expand. Fig 8B and 8D show the number of communities in the optimal partition obtained using the Leiden algorithm. Here, there is no clear relationship between the size of the network and the number of communities. In Fig 8B, we can see that hsCPP has many more communities when built at *p* = 20% than at 40%. In contrast, esSC has the same number of communities regardless of the percentage of nodes included. This pattern is commonly observed, as community detection algorithms do not fragment networks based on their number of nodes but rather on the configuration of their links.

Notably, changes in signed modularity do not necessarily translate into changes in the number of detected communities, highlighting that these two measures capture complementary aspects of mesoscale organization in directed signed networks. Importantly, attack and defense networks again exhibit very similar values, by showing that these two subnetworks display comparable community structure despite being constructed from distinct sets of negative hubs.

## Discussion

The study of student relationships within school systems has received much attention from researchers. Kucharski et al. investigate face-to-face relationship networks in secondary schools and analyze how these change over time [39]. Boda et al. examine how ethnic factors influence personal relationships in a signed network of a secondary school [40]. Meanwhile, in reference [41], the authors study the temporal evolution of networks in elementary schools using face-to-face proximity sensors. Within this line of research, bullying is a phenomenon of great interest. Salmivalli’s work examines this phenomenon as both group- and individual- level processes, highlighting that prevention policies and individuals’ motivations must be in line with behaviors that promote it [42]. In reference [43], bullying and its negative consequences (anxiety, insomnia, and depression) on the health and academic performance of children in elementary schools are analyzed.

Kisfalusi et al. conducted a longitudinal study in fifteen elementary schools in the Netherlands. They defined bullying as repeated aggressive behavior directed at less powerful individuals, while dislike relationships were characterized by negative feelings toward another person. Their findings indicate that dislike relationships frequently escalate into bully-victim dynamics over time. Additionally, the authors examined negative hubs (they call them dislike childrens), demonstrating that these individuals are more likely to be involved in bully-victim dynamics, supporting a conceptual link between negative relationship and bullying-related roles [22]. Similarly, Wittek et al., in a longitudinal study, examined the interplay between enmity relationships and physical violence among students, highlighting the role of negative ties in shaping the social structure of conflict [21]. These studies support the idea that dislike or negative ties can reflect underlying social tensions that may be related to more explicit forms of aggression or exclusion among peers.

In our study, we acknowledge that the applied surveys capture dislike relationships. However, studies such as those mentioned above show that dislike networks are meaningfully connected to bully-victim dynamics. The findings of our research are relevant to the study of directed signed networks and gain further significance by providing a structural framework to conceptually interpret out-negative hubs, in-negative hubs, and i/o-negative hubs in relation to bullying-related roles, such as bullies, victims, and aggressive victims.

It is important to clarify that, although negative or dislike relationships can be related to bullying dynamics, our dataset does not provide direct behavioral evidence of bullying. Therefore, throughout this work we interpret negative links as structural indicators of social tension or conflict rather than explicit acts of bullying. The references to bullying are provided as a theoretical framework to contextualize the potential implications of negative relationships in school environments, in line with previous studies that have associated dislike networks with bully-victim relationships.

From this broader perspective, international reports indicate that bullying and school violence remain prevalent phenomena in educational settings. According to UNESCO’s 2023 report, approximately one-third of children and adolescents worldwide experience school bullying, with gender disparities varying by region. While boys generally face higher bullying rates overall, girls show greater vulnerability in high-incidence countries like México [44,45]. Consistent with this pattern, our attack and defense networks include a proportionally higher number of female students. The UNESCO report further distinguishes bullying types: physical aggression predominates among boys, while psychological bullying more commonly targets girls. In addition, the prevalence of bullying tends to decrease with age. In our study, we observe a parallel descriptive pattern in the network structure, where negative relationships in attack and defense networks decline at higher academic levels (Figs 2, 4, 6).

### Aggressive victims

From a social perspective, bullies and victims have traditionally been treated as mutually exclusive roles, with distinct methodological approaches used to study each group [46]. However, some research acknowledges the existence of individuals who simultaneously occupy both roles, commonly referred to as aggressive victims [47–49].

Del Moral et al. emphasize the significant social role of aggressive victims in school environments, noting their distinct behavioral patterns and the varying levels of violent responses to victimization [50]. Previous studies have described aggressive victims as more reactive and prone to risky behaviors [47]. Olweus suggests that aggressive victims respond to provocation with extreme hostility, in contrast to passive victims, who exhibit submissive behaviors [51]. Furthermore, aggressive victims tend to have few friends [47], which may help explain why they often remain undetected in studies focusing exclusively on positive ties, where they tend to occupy peripheral and seemingly insignificant network positions.

From a network perspective, a structurally analogous pattern emerges in our results. The i/o-negative hubs (the most negatively connected nodes, with high negative reciprocity) persist in both attack and defense networks and help to maintain their topological similarity. This suggests their structural importance in directed signed networks.

In this study, we conceptually relate i/o-negative hub nodes to aggressive victims, although it is likely that not all of these nodes fall into that category. Olweus reported that approximately 20% of victims exhibited aggressive victim characteristics [51], a proportion lower than the values observed in our networks (Fig 3A). This difference is expected, as behavioral classifications identify specific individuals, whereas our approach captures structurally defined roles based on patterns of negative reciprocity. More recent literature has proposed broader conceptualizations of aggressive victims [50], identifying multiple subtypes within this category, which may lead to prevalence estimates closer to those observed in structurally defined analyses. At the network level, these findings emphasize the relevance of i/o-negative hubs as structurally central nodes in antagonistic relationship systems, regardless of their exact behavioral classification.

### Gender-based affinities and conflicts

Kisfalusi et al. suggest that bullying and dislike relationships are predominantly formed between same-gender individuals, and that negative interactions play a particularly salient role within these group dynamics [22]. Consistent with this literature, we observe higher $\langle k\rangle^{-}$ values for same-gender connections in both attack and defense networks (Fig 6). This pattern of gender homophily in negative relationships is further supported by the predominance of same-gender positive links (see Fig G in S1 Fig), suggesting that negative hubs tend to interact more frequently with same-gender peers. Furthermore, Fig 6A and 6C show that in elementary schools (esSC, esRRC, and esIZ) cross-gender negative relationships are more frequent, a pattern that decreases at higher academic levels, consistent with the progressive emergence of gender homophily reported in previous studies.

Hooijsma et al. [52] examine gender-specific patterns in aggressor-victim friendship relationships, finding that bullies tend to form more same-gender friendships when they are targeting the same victim. Our analysis of attack and defense networks by relationship type (see Fig G in S1 Fig) shows higher $\langle k\rangle^{+}$ values among same-gender students compared to cross-gender students. From a network perspective, this pattern is consistent with a greater tendency toward coordinated social contexts among same-gender students, however, we do not have direct evidence of shared victimization.

Ettekal et al. [49] document gender differences in aggressive victim behavior: females predominantly engage in relational aggression (non-physical behaviors targeting social status or reputation) and experience relational victimization more frequently, while males exhibit primarily physical and verbal aggression. The authors also suggest that relationally aggressive female victims often maintain larger friendship networks, which gives them greater social influence to perpetuate harm. At a structural level, these behavioral patterns may help interpret the observed differences in $\langle k\rangle^{+}$ values when comparing attack and defense networks by gender (see Fig E in S1 Fig), where higher values are found among female students.

These patterns are descriptive and emphasize structural tendencies rather than statistically tested differences. This approach allows us to identify meaningful patterns of gender homophily and conflict without overinterpreting the observed differences as statistically significant effects.

### Social balance and communities

We adopted the methodology proposed by Aref et al., in which social balance is measured by evaluating four types of triads, those with the highest number of transitive semi-cycles [7]. The social balance values obtained for the attack and defense networks are consistently high across schools and node-selection thresholds (see Fig 7). The values fall within the range commonly reported for empirical signed social networks, which tend to exhibit higher levels of social balance than those expected under random models [7]. Despite being constructed based on negative hubs, the attack and defense networks can exhibit relatively high levels of social balance despite their antagonistic nature.

Complementing these findings, Salmivalli [42] documents that bullies are often supported by assistants (co-perpetrators) within peer groups, reinforcing aggressive behavior. Structurally, the presence of well-defined communities in the attack and defense networks (see Fig 8) suggests that antagonistic relationships are embedded within cohesive mesoscale structures. This structural organization is consistent with the group-based dynamics described by Salmivalli, although our data do not allow us to identify specific behavioral roles.

Finally, it is important to acknowledge that our analyses are conditioned on the selection of nodes with the highest negative connectivity (negative hubs), which may introduce boundary effects in the estimation of social balance and communities. To ensure robustness, we evaluated the stability of the results across the 500 stochastic replications performed for each (school, *p*) condition and verified the consistency of social balance and communities values across the analyzed percentage range p∈[20%,40%]. These checks confirmed that the observed trends remain stable within this interval.

### Limitations of the study

- Although our discussions involve concepts related to bullying and victimization, the data are limited to students’ reports of positive and negative relationships. As such, the study cannot directly identify bullying behaviors, but rather captures structural patterns of negative social relationships.
- As we mentioned before, since our analyses are conditioned on subnetworks constructed from the most negatively connected nodes, some boundary effects may arise. Additionally, the subnetwork construction conditions on the selection of negative hubs, which may introduce structural biases. By focusing only on nodes with high negative degree, the resulting subnetworks reflect a subset of the social system, and their properties may differ from those of the full network.
- The data were collected from schools in a single region of México, which may limit the generalizability of the findings to other cultural or institutional contexts.
- The cross-sectional design does not allow us to examine temporal dynamics or make causal claims regarding the emergence of negative or positive ties. Longitudinal data would be necessary to evaluate how attack and defense relations evolve over time.

### Future works

- Future studies could combine signed network data with validated behavioral measures to assess how structurally defined roles relate to bullying-related roles.
- Previous studies have shown that hub nodes play an important role in the structural robustness of complex networks [53]. Future work could examine how i/o-negative hubs influence the robustness of the original networks through targeted node removal, for example by evaluating changes in the size of the largest connected component and network fragmentation.
- This study shows that the structure formed by the i/o-negative hubs preserves similar topological properties between the attack and defense networks. It would be of great interest to verify whether these properties remain consistent when compared to larger networks, for instance, the strongly connected components or the largest connected components of the original networks.
- This study was approached from the perspective of negative hubs; however, a similar methodology could be applied focusing on positive hubs to examine similarities and differences between both cases.

### Additional notes

In their study, Oldenburg et al. previously used the term ’defending networks’. Students who had experienced at least one form of bullying in recent months were asked to nominate their defenders, that is, classmates who supported, comforted, or helped them when they were bullied. In this way, the authors created ego-networks centered around the victimized student, which they referred to as defending networks [54]. In contrast, the ’defense networks’ analyzed here are defined purely in structural terms, as subnetworks centered on nodes with high in-negative degree, and do not represent defending actions or roles.

## Conclusion

In this study, we examined directed signed networks that represent the relationships among students from nine schools at different academic levels. We defined attack networks and defense networks and analyzed the importance of the most negatively connected nodes. We observed that, when comparing attack and defense networks, they maintain similar topological properties (average degrees, social balance, and modularity), and this similarity is consistent with the structure of the i/o-negative hubs. We define i/o-negative hubs as those nodes with high in-negative and out-negative degree values.

By calculating the social balance and modularity of the attack and defense networks, relatively high values were observed; this indicates that, despite being built around negative hubs, these networks maintain coherent and stable structural patterns. We also conducted an analysis based on the gender of the students, finding that female attack and defense networks tended to be denser in terms of negative relationships, and same-gender negative ties were generally more prevalent than cross-gender negative ties, particularly at higher academic levels. We conceptually relate i/o-negative hubs to aggressive victims, who have been described in the literature as individuals who may generate more conflict due to reactive patterns of behavior.

The methodology proposed in this study provides a structural framework for analyzing negative relationships in directed signed networks and may help to conceptually interpret patterns associated with bullying-related roles in other contexts. Bullying is a highly complex dynamic and a globally impactful issue, and improving our understanding of the structural organization of negative relationships may be useful for future research and prevention frameworks. Overall, the findings of this study contribute to a better understanding of directed signed networks and their usefulness for analyzing complex social systems through friendship and enmity relationships.

## Supporting information

S1 File Questionnaires. Questions asked in data collection (english and spanish versions).
(PDF)

S1 Dataset Adjacency Matrix. Copies of the nine signed adjacency matrices used in the study.
(ZIP)

S2 Dataset Node-level data. Anonymized node-level dataset including node id, gender, and classroom membership for all schools analyzed.
(ZIP)

S1 Fig Support Figures. Additional visualizations supporting main analyses.
(PDF)
