# Supplementary material for: Attack and defense networks in a student social system
Source: PLoS One. 2026 May 18;21(5):e0348814. doi: 10.1371/journal.pone.0348814 (PMC13183207; doi:10.1371/journal.pone.0348814)
Supplement: S1 Fig — Additional visualizations supporting main analyses. (PDF) [file pone.0348814.s004.pdf]

Attack and defense networks in a student social system.

Alejandro Morales-Huitrón[1], Ana María Hernández-Hernández[2], Efrain Canto-Lugo[2*], Rodrigo Huerta-Quintanilla[2].

1 Departamento de Investigación y Estudios Multidisciplinarios, Centro de Investigación y de Estudios Avanzados del Instituto Politécnico Nacional. Unidad Zacatenco, Gustavo A. Madero, Ciudad de México 07360, México.
2 Departamento de Física Aplicada, Centro de Investigación y de Estudios Avanzados del Instituto Politécnico Nacional. Unidad Mérida, Mérida, Yucatán 97310, México.

* ecanto@cinvestav.mx

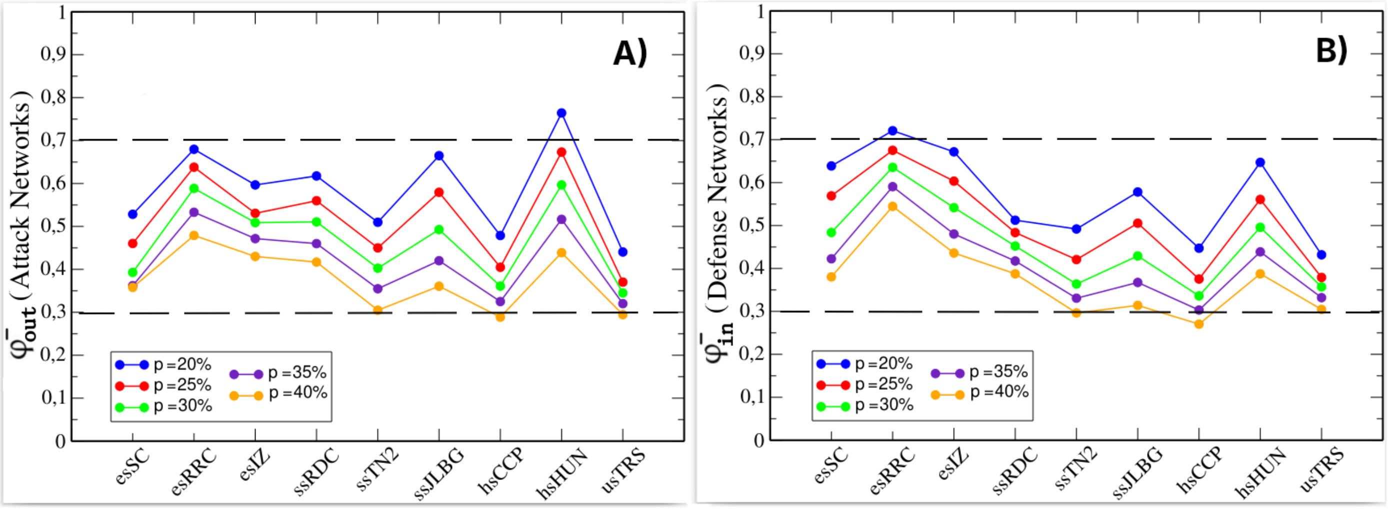

Fig A. Structural equilibrium. A) Structural equilibrium in attack networks. B) Structural equilibrium in defense networks. Color scale indicates percentage of nodes (p) included from the original network. Each value was obtained after 500 simulations.

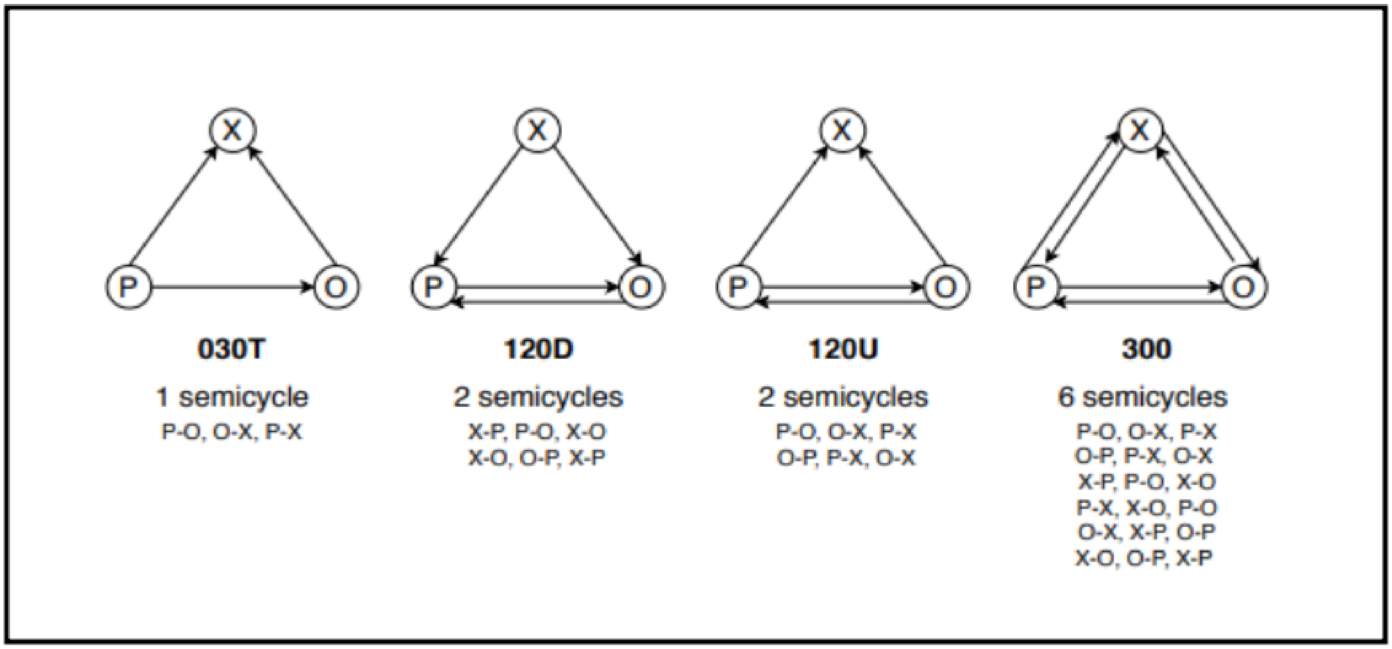

Fig B. Transitive triads Transitive triads with the nomenclature 030T, 120D, 120U, and 300, and their respective semi-cycles. Image taken from Structural balance in real-world social networks: incorporating direction and transitivity in measuring partial balance. doi.org/10.1007/s13278-024-01339-1

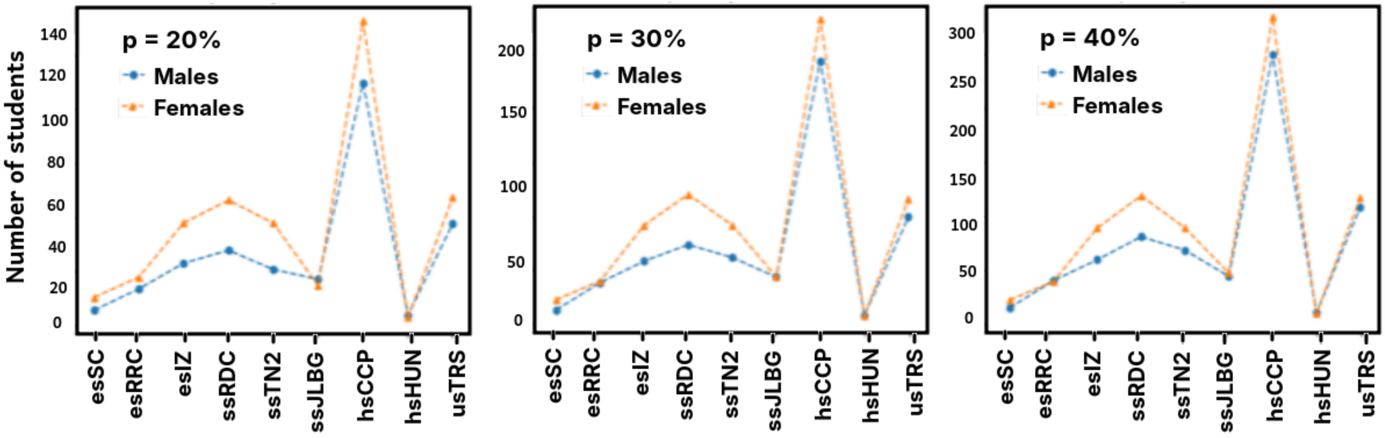

Fig C. Number of students by gender in the attack networks. The p-value represents the percentage of nodes included from the original network. Each value was obtained after 500 simulations.

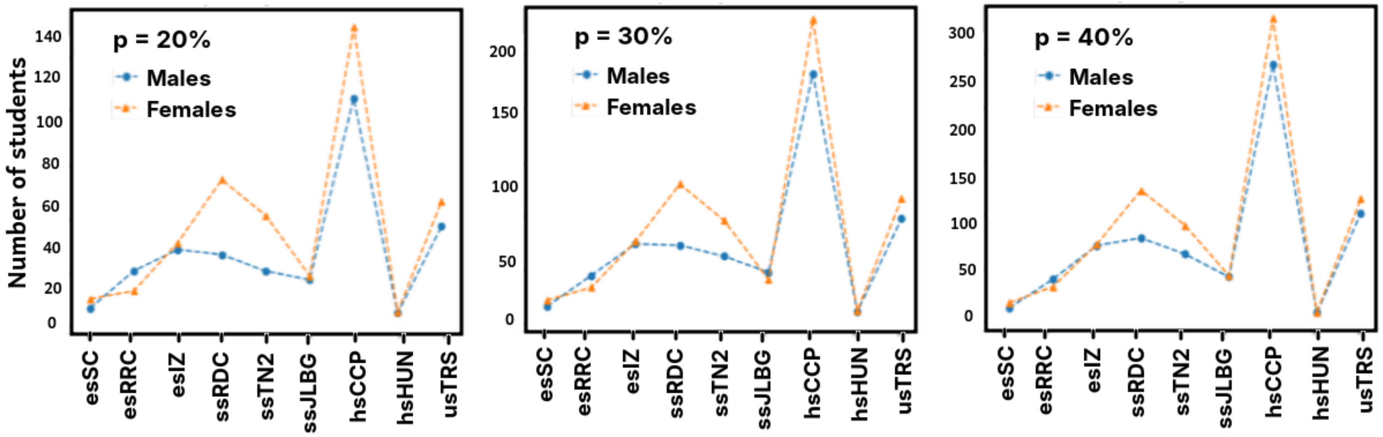

Fig D. Number of students by gender in the defense networks. The p-value represents the percentage of nodes included from the original network. Each value was obtained after 500 simulations.

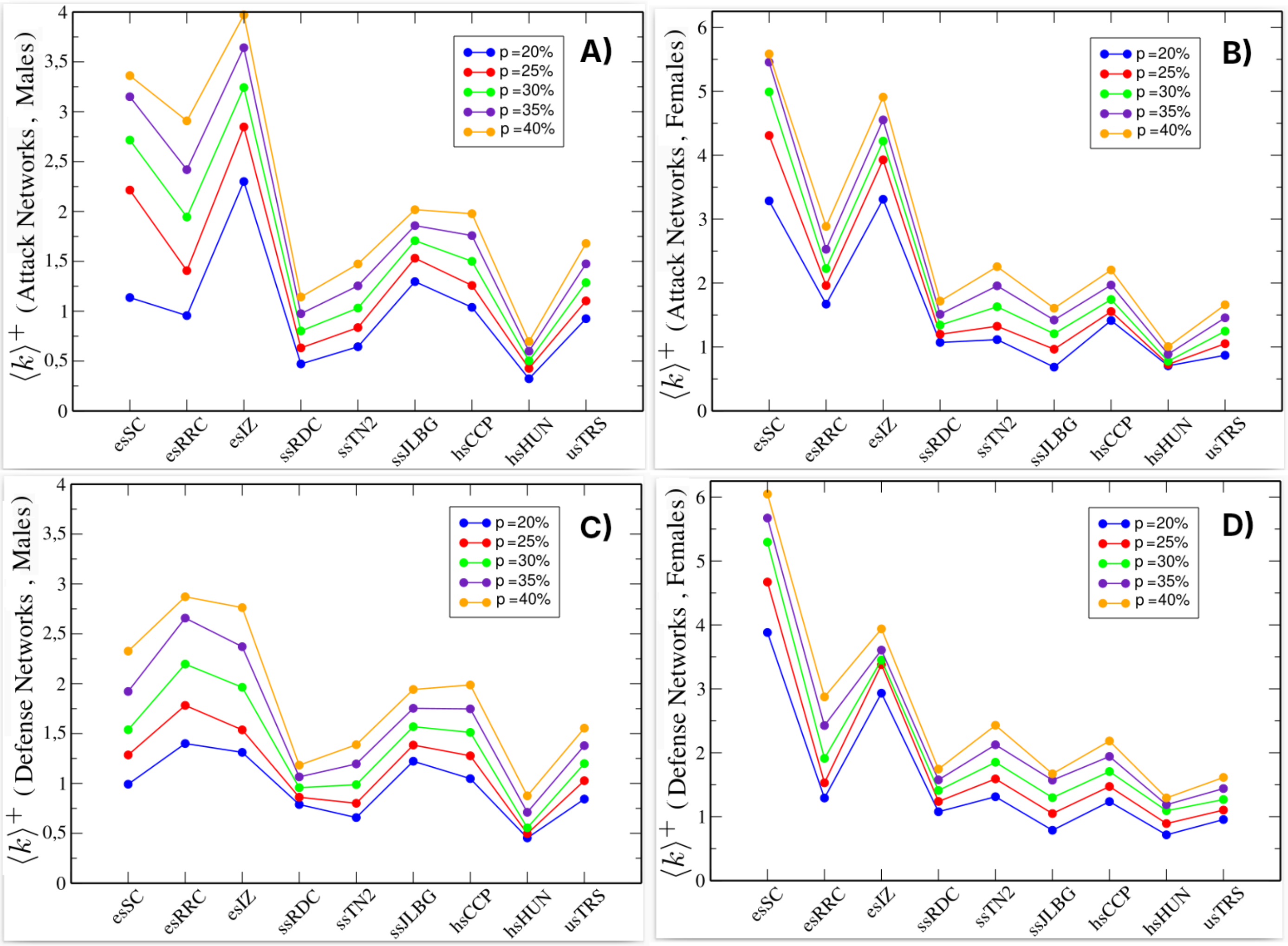

Fig E. Average positive degree in attack and defense networks by gender (A) Male attack networks (male out-negative hubs). (B) Female attack networks (female out-negative hubs). (C) Male defense networks (male in-negative hubs). (D) Female defense networks (female in-negative hubs). Color scale indicates percentage of nodes (p) included from the original network. Each value was obtained after 500 simulations.

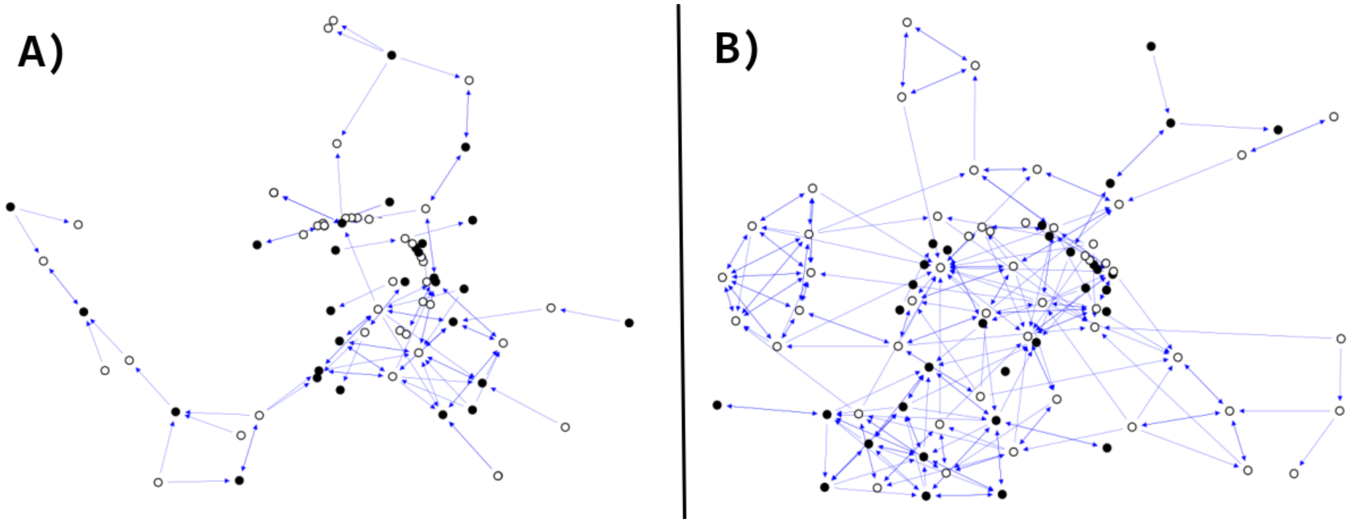

Fig F. Positive link networks by relationship type. (A) Attack network of school esIZ at $p = 20\%$. This network contains 81 nodes, of which 31 males and 50 females. Only positive relationships connecting cross-gender nodes are shown. (B) Defense network of school esIZ at $p = 20\%$. This network contains 82 nodes, of which 31 males and 51 females. Only positive relationships connecting same-gender nodes are shown. The color of the nodes color indicates the gender: males (black) and females (white). Note: The network in panel B has two disconnected components, as there are no links between male and female nodes.

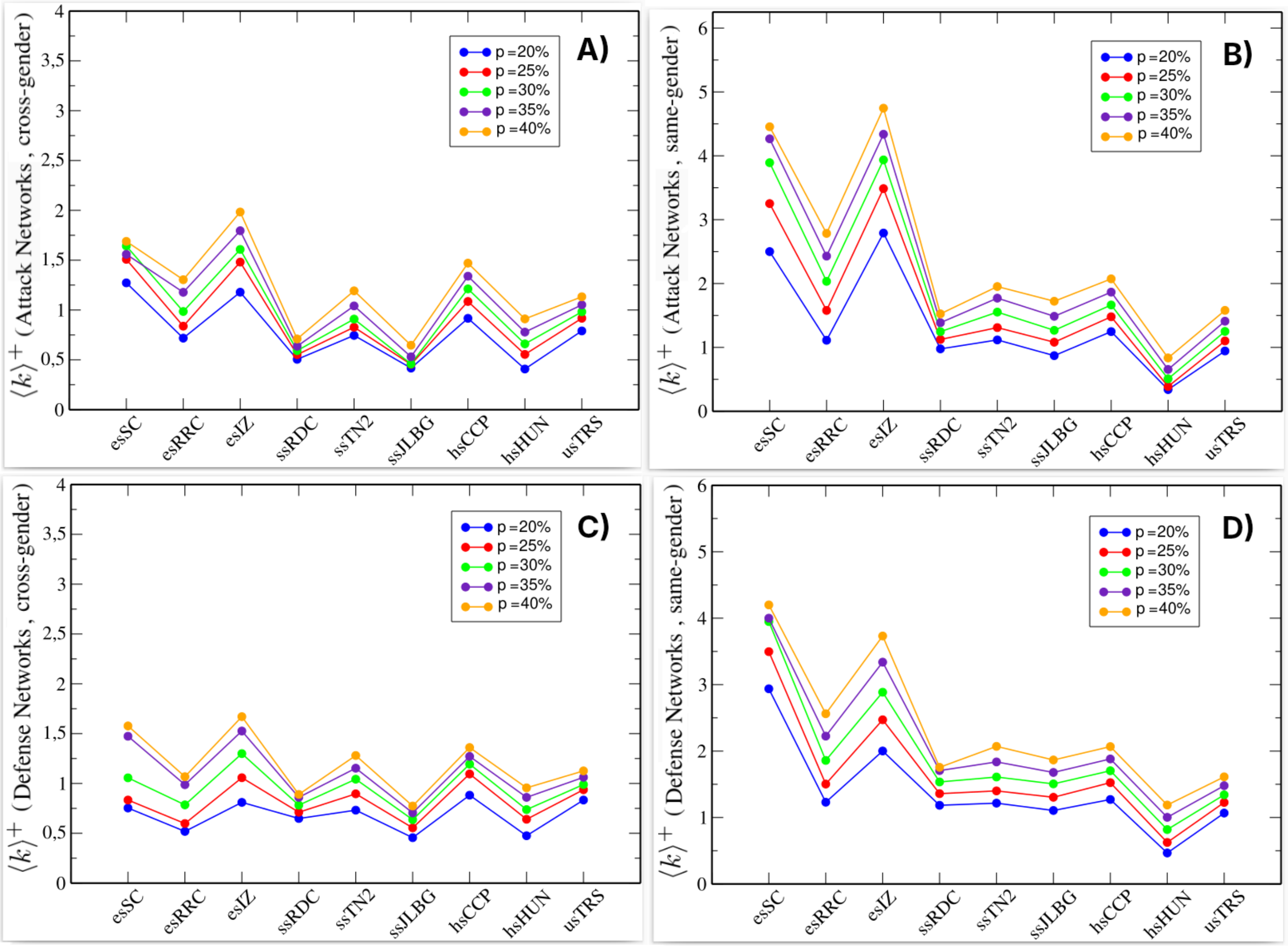

Fig G. Average degree of positive links in attack and defense networks by relationship type. (A) Attack networks with relationships between cross-gender students. (B) Attack networks with relationships between same-gender students. (C) Defense networks with relationships between cross-genders students. (D) Defense networks with relationships between same-gender students. Color scale indicates percentage of nodes (p) included from the original network. Each value was obtained after 500 simulations.
